# *N*-[(2*S*)-2-Chloro­propano­yl]glycine

**DOI:** 10.1107/S1600536812036720

**Published:** 2012-08-31

**Authors:** Hong-shun Sun, Yi Tang, Yu-long Li, Ning Xu, Hong Xu

**Affiliations:** a Chemical Engineering Department, Nanjing College of Chemical Technology, Geguan Road No. 265 Nanjing, Nanjing 210048, People’s Republic of China; b Anhui University of Architecture, Ziyun Road No. 292, Economic Development Zone, Hefei City, Hefei 210048, People’s Republic of China

## Abstract

The title compound, C_5_H_8_ClNO_3_, was prepared by the nucleophilic substitution reaction of (2*S*)-2-chloro­propanoyl chloride with glycine. The acetate group forms a dihedral angle of 84.6 (1)° with the mean plane of the C—NH—C=O fragment. In the crystal, the molecules are linked by N—H⋯O and O—H⋯O hydrogen bonds, generating a three-dimensional network, which consolidates the crystal packing.

## Related literature

The title compound is an inter­mediate of Tiopronin [systematic name: *N*-(2-sulfanyl­propano­yl)glycine], a prescription thiol drug used to control the rate of cystine precipitation and excretion in the disease cystinuria, see: Wang *et al.* (1993 ▶). For a related structure, see: Lv *et al.* (2007 ▶).
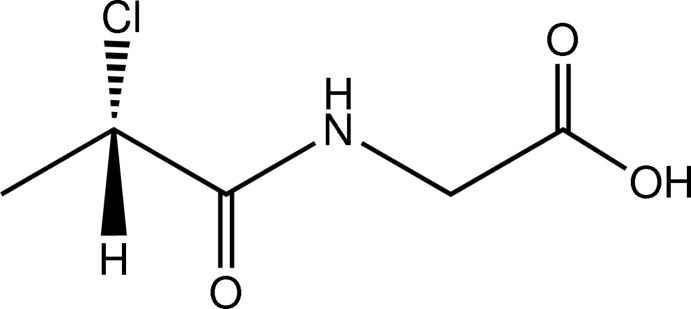


## Experimental

### 

#### Crystal data

C_5_H_8_ClNO_3_
*M* *_r_* = 165.57
Orthorhombic, 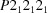

*a* = 5.5170 (11) Å
*b* = 11.622 (2) Å
*c* = 11.964 (2) Å
*V* = 767.1 (3) Å^3^
*Z* = 4
Mo *K*α radiation
μ = 0.45 mm^−1^
*T* = 293 K
0.30 × 0.20 × 0.10 mm

#### Data collection

Enraf–Nonius CAD-4 diffractometer
Absorption correction: ψ scan (North *et al.* , 1968 ▶) *T*_min_ = 0.878, *T*_max_ = 0.957
1630 measured reflections
1413 independent reflections
1283 reflections with *I* > 2σ(*I*)
*R*_int_ = 0.024
3 standard reflections every 200 reflections intensity decay: 1%

#### Refinement

*R*[*F* ^2^ > 2σ(*F* ^2^)] = 0.035
*wR*(*F* ^2^) = 0.100
*S* = 1.00
1413 reflections
91 parameters
H-atom parameters constrained
Δρ_max_ = 0.22 e Å^−3^
Δρ_min_ = −0.21 e Å^−3^
Absolute structure: Flack (1983 ▶), 545 Friedel pairs
Flack parameter: 0.19 (9)

### 

Data collection: *CAD-4 EXPRESS* (Enraf–Nonius, 1994 ▶); cell refinement: *CAD-4 EXPRESS*; data reduction: *XCAD4* (Harms & Wocadlo,1995 ▶); program(s) used to solve structure: *SHELXTL* (Sheldrick, 2008 ▶); program(s) used to refine structure: *SHELXTL*; molecular graphics: *SHELXTL*; software used to prepare material for publication: *SHELXTL*.

## Supplementary Material

Crystal structure: contains datablock(s) I, global. DOI: 10.1107/S1600536812036720/cv5331sup1.cif

Structure factors: contains datablock(s) I. DOI: 10.1107/S1600536812036720/cv5331Isup2.hkl

Supplementary material file. DOI: 10.1107/S1600536812036720/cv5331Isup3.cml

Additional supplementary materials:  crystallographic information; 3D view; checkCIF report

## Figures and Tables

**Table 1:** Hydrogen-bond geometry (Å, °)

*D*—H⋯*A*	*D*—H	H⋯*A*	*D*⋯*A*	*D*—H⋯*A*
N1—H1⋯O2^i^	0.86	2.09	2.920 (3)	161
O3—H3*A*⋯O1^ii^	0.85	1.79	2.629 (3)	172

Symmetry codes: (i) 
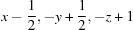
; (ii) 
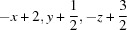
.

**Fig. 1.**
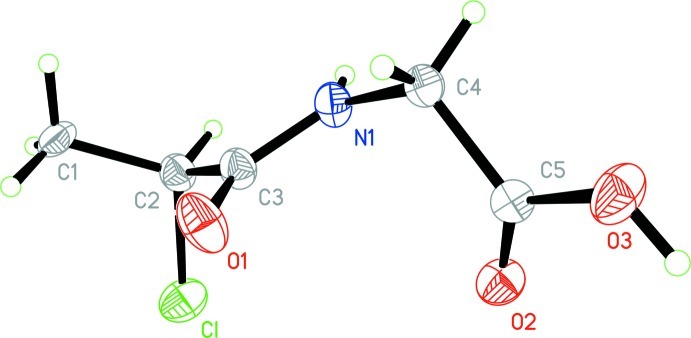
The molecular structure of (I), showing the atom-numbering scheme and displacement ellipsoids at the 30% probability level.

## supplementary crystallographic information

### Comment

The title compound, (I), is an important intermediate in the synthesis of Tiopronin (Wang *et al.*, 1993), which is a prescription thiol drug used to control the rate of cystine precipitation and excretion in the disease cystinuria. Herewith we report the synthesis and the crystal structure of (I).

In the molecule of (I) (Fig. 1), the bond lengths and angles are within normal ranges and correspond to those observed in the related compound (Lv *et al.*, 2007). Atoms C2, C3, O1, N and C4 are nearly coplanar, with a dihedral angle of 0.7 (3)° between the C2/C3/O1 and O1/C3/N/C4 planes, the acetate group forms a dihedral angle of 84.6 (1)° with the mean plane of C4—N1(H1)—C3═O1 fragment. The quiral atom C2 shows an S absolute configuration. Intermolecular N—H···O and O—H···O hydrogen bonds (Table 1) link the molecules into a three-dimensional network, which consolidate the crystal packing.

### Experimental

An aqueous solution of 120 g (1.6 mole) of glycine and 84.8 g (0.8 mole) of sodium carbonate is placed in a 1*L* four-necked flask fitted with a mechanical stirrer and two dropping funnels. The flask is cooled in an ice bath, and 203.2 g (1.6 mol) of (2*S*)-2-chloropropanoyl chloride and 400 ml of 4 N sodium carbonate are added simultaneously to the vigorously stirred solution over a period of 20–25 minutes. The mixture is stirred for an additional 3 h. The aqueous solution is cooled in an ice bath and acidified to Congo red with concentrated hydrochloric acid.The title compound was extracted from the reaction mixture using ethyl acetate and subsequently crystallized from the same solvent (yield 212 g, 80%; m.p. 377–378 K).

### Refinement

H atoms were positioned geometrically, with O—H = 0.85 Å, N— H = 0.86 Å and C—H = 0.96, 0.97 and 0.98 Å for methyl, methylene and methine H, respectively, and constrained to ride on their parent atoms, with *U*_iso_(H) = xUeq(C,*N*,*O*), where *x* = 1.5 for OH and methyl H, and *x* = 1.2 for all other H atoms.

### Crystal data

**Table:**

C_5_H_8_ClNO_3_	*F*(000) = 344
*M**_r_* = 165.57	*D*_x_ = 1.434 Mg m^−^^3^
Orthorhombic, *P*2_1_2_1_2_1_	Mo *K*α radiation, λ = 0.71073 Å
Hall symbol: P 2ac 2ab	Cell parameters from 25 reflections
*a* = 5.5170 (11) Å	θ = 9–13°
*b* = 11.622 (2) Å	µ = 0.45 mm^−^^1^
*c* = 11.964 (2) Å	*T* = 293 K
*V* = 767.1 (3) Å^3^	Block, colourless
*Z* = 4	0.30 × 0.20 × 0.10 mm

### Data collection

**Table:**

Enraf–Nonius CAD-4 diffractometer	1283 reflections with *I* > 2σ(*I*)
Radiation source: fine-focus sealed tube	*R*_int_ = 0.024
Graphite monochromator	θ_max_ = 25.4°, θ_min_ = 2.4°
ω/2θ scans	*h* = 0→6
Absorption correction: ψ scan (North *et al.* , 1968)	*k* = 0→14
*T*_min_ = 0.878, *T*_max_ = 0.957	*l* = −14→14
1630 measured reflections	3 standard reflections every 200 reflections
1413 independent reflections	intensity decay: 1%

### Refinement

**Table:**

Refinement on *F*^2^	Secondary atom site location: difference Fourier map
Least-squares matrix: full	Hydrogen site location: inferred from neighbouring sites
*R*[*F*^2^ > 2σ(*F*^2^)] = 0.035	H-atom parameters constrained
*wR*(*F*^2^) = 0.100	*w* = 1/[σ^2^(*F*_o_^2^) + (0.0769*P*)^2^] where *P* = (*F*_o_^2^ + 2*F*_c_^2^)/3
*S* = 1.00	(Δ/σ)_max_ < 0.001
1413 reflections	Δρ_max_ = 0.22 e Å^−^^3^
91 parameters	Δρ_min_ = −0.21 e Å^−^^3^
0 restraints	Absolute structure: Flack (1983), 545 Friedel pairs
Primary atom site location: structure-invariant direct methods	Flack parameter: 0.19 (9)

### Special details

**Table:**

Geometry. All e.s.d.'s (except the e.s.d. in the dihedral angle between two l.s. planes) are estimated using the full covariance matrix. The cell e.s.d.'s are taken into account individually in the estimation of e.s.d.'s in distances, angles and torsion angles; correlations between e.s.d.'s in cell parameters are only used when they are defined by crystal symmetry. An approximate (isotropic) treatment of cell e.s.d.'s is used for estimating e.s.d.'s involving l.s. planes.
Refinement. Refinement of *F*^2^ against ALL reflections. The weighted *R*-factor *wR* and goodness of fit *S* are based on *F*^2^, conventional *R*-factors *R* are based on *F*, with *F* set to zero for negative *F*^2^. The threshold expression of *F*^2^ > σ(*F*^2^) is used only for calculating *R*-factors(gt) *etc*. and is not relevant to the choice of reflections for refinement. *R*-factors based on *F*^2^ are statistically about twice as large as those based on *F*, and *R*- factors based on ALL data will be even larger.

### Fractional atomic coordinates and isotropic or equivalent isotropic displacement parameters (Å^2^)

**Table:**

	*x*	*y*	*z*	*U*_iso_*/*U*_eq_	
Cl	1.07098 (13)	−0.02109 (7)	0.44996 (6)	0.0614 (3)	
N1	0.6201 (3)	0.14605 (16)	0.59648 (15)	0.0380 (4)	
H1	0.5455	0.1604	0.5349	0.046*	
O1	0.8581 (4)	0.02471 (16)	0.69152 (14)	0.0539 (5)	
C1	0.6872 (4)	−0.1485 (2)	0.5215 (2)	0.0428 (5)	
H1A	0.5231	−0.1506	0.5481	0.064*	
H1B	0.7926	−0.1819	0.5765	0.064*	
H1C	0.6990	−0.1914	0.4531	0.064*	
O2	0.9834 (3)	0.30550 (15)	0.63454 (14)	0.0454 (4)	
C2	0.7635 (4)	−0.02114 (18)	0.49985 (19)	0.0400 (5)	
H2A	0.6573	0.0126	0.4429	0.048*	
O3	0.7988 (3)	0.36913 (16)	0.78778 (13)	0.0510 (5)	
H3A	0.9157	0.4167	0.7885	0.077*	
C3	0.7545 (4)	0.05143 (17)	0.60461 (17)	0.0358 (5)	
C4	0.5972 (4)	0.22508 (19)	0.68892 (17)	0.0348 (5)	
H4A	0.5776	0.1818	0.7577	0.042*	
H4B	0.4532	0.2718	0.6785	0.042*	
C5	0.8171 (4)	0.30310 (18)	0.69913 (16)	0.0319 (4)	

### Atomic displacement parameters (Å^2^)

**Table:**

	*U*^11^	*U*^22^	*U*^33^	*U*^12^	*U*^13^	*U*^23^
Cl	0.0554 (4)	0.0715 (5)	0.0574 (4)	−0.0122 (3)	0.0123 (3)	0.0006 (3)
N1	0.0455 (11)	0.0322 (9)	0.0364 (9)	0.0033 (8)	−0.0140 (8)	−0.0019 (8)
O1	0.0702 (12)	0.0506 (9)	0.0408 (9)	0.0207 (8)	−0.0128 (8)	0.0012 (8)
C1	0.0344 (11)	0.0356 (11)	0.0584 (14)	−0.0128 (10)	0.0106 (11)	−0.0106 (11)
O2	0.0451 (9)	0.0525 (10)	0.0386 (8)	−0.0076 (8)	0.0090 (7)	−0.0003 (7)
C2	0.0417 (11)	0.0370 (11)	0.0412 (12)	0.0026 (10)	−0.0048 (10)	−0.0003 (9)
O3	0.0569 (10)	0.0583 (10)	0.0379 (8)	−0.0186 (9)	0.0058 (8)	−0.0159 (8)
C3	0.0391 (11)	0.0322 (10)	0.0361 (11)	0.0026 (9)	−0.0079 (10)	0.0003 (8)
C4	0.0354 (11)	0.0333 (10)	0.0358 (11)	0.0008 (9)	0.0013 (9)	−0.0033 (9)
C5	0.0359 (10)	0.0357 (10)	0.0240 (9)	−0.0009 (9)	0.0000 (9)	0.0036 (8)

### Geometric parameters (Å, º)

**Table:**

Cl—C2	1.798 (2)	O2—C5	1.200 (3)
N1—C3	1.330 (3)	C2—C3	1.512 (3)
N1—C4	1.443 (3)	C2—H2A	0.9800
N1—H1	0.8600	O3—C5	1.313 (3)
O1—C3	1.227 (3)	O3—H3A	0.8500
C1—C2	1.561 (3)	C4—C5	1.520 (3)
C1—H1A	0.9600	C4—H4A	0.9700
C1—H1B	0.9600	C4—H4B	0.9700
C1—H1C	0.9600		
			
C3—N1—C4	121.28 (18)	Cl—C2—H2A	109.5
C3—N1—H1	119.4	C5—O3—H3A	109.3
C4—N1—H1	119.4	O1—C3—N1	122.1 (2)
C2—C1—H1A	109.5	O1—C3—C2	123.12 (18)
C2—C1—H1B	109.5	N1—C3—C2	114.78 (18)
H1A—C1—H1B	109.5	N1—C4—C5	111.79 (18)
C2—C1—H1C	109.5	N1—C4—H4A	109.3
H1A—C1—H1C	109.5	C5—C4—H4A	109.3
H1B—C1—H1C	109.5	N1—C4—H4B	109.3
C3—C2—C1	112.52 (19)	C5—C4—H4B	109.3
C3—C2—Cl	107.82 (15)	H4A—C4—H4B	107.9
C1—C2—Cl	108.03 (15)	O2—C5—O3	124.4 (2)
C3—C2—H2A	109.5	O2—C5—C4	124.94 (19)
C1—C2—H2A	109.5	O3—C5—C4	110.62 (17)
			
C4—N1—C3—O1	2.5 (3)	Cl—C2—C3—N1	115.56 (18)
C4—N1—C3—C2	−178.9 (2)	C3—N1—C4—C5	79.1 (3)
C1—C2—C3—O1	53.1 (3)	N1—C4—C5—O2	4.9 (3)
Cl—C2—C3—O1	−65.9 (3)	N1—C4—C5—O3	−176.05 (18)
C1—C2—C3—N1	−125.4 (2)		

### Hydrogen-bond geometry (Å, º)

**Table:**

*D*—H···*A*	*D*—H	H···*A*	*D*···*A*	*D*—H···*A*
N1—H1···O2^i^	0.86	2.09	2.920 (3)	161
O3—H3*A*···O1^ii^	0.85	1.79	2.629 (3)	172

Symmetry codes: (i) *x*−1/2, −*y*+1/2, −*z*+1; (ii) −*x*+2, *y*+1/2, −*z*+3/2.

## References

[bb1] Enraf–Nonius (1994). *CAD-4 EXPRESS* Enraf–Nonius, Delft, The Netherlands.

[bb2] Flack, H. D. (1983). *Acta Cryst.* A**39**, 876–881.

[bb3] Harms, K. & Wocadlo, S. (1995). *XCAD4* University of Marburg, Germany.

[bb4] Lv, Z.-F., Gao, X.-S., Wu, W.-Y., Gao, X.-F. & Wang, J.-T. (2007). *Acta Cryst.* E**63**, o485–o486.

[bb5] North, A. C. T., Phillips, D. C. & Mathews, F. S. (1968). *Acta Cryst.* A**24**, 351–359.

[bb6] Sheldrick, G. M. (2008). *Acta Cryst.* A**64**, 112–122. 10.1107/S0108767307043930 18156677

[bb7] Wang, D.-Y., Zhang, C.-Z., Liu, J. & Shi, W.-H. (1993). *Zhongguo Yiyao Gongye Zazhi*, **24**, 243.

